# Inhibition of EZH2 by chemo- and radiotherapy agents and small molecule inhibitors induces cell death in castration-resistant prostate cancer

**DOI:** 10.18632/oncotarget.6497

**Published:** 2015-12-07

**Authors:** Changping Wu, Xin Jin, Jing Yang, Yinhui Yang, Yundong He, Liya Ding, Yunqian Pan, Shuai Chen, Jingting Jiang, Haojie Huang

**Affiliations:** ^1^ Department of Tumor Biological Treatment, The Third Affiliated Hospital of Soochow University, Changzhou 213003, China; ^2^ Department of Biochemistry and Molecular Biology, Mayo Clinic College of Medicine, Rochester, MN 55905, USA; ^3^ Department of Urology, Mayo Clinic College of Medicine, Rochester, MN 55905, USA; ^4^ Mayo Clinic Cancer Center, Mayo Clinic College of Medicine, Rochester, MN 55905, USA; ^5^ Sun Yat-sen University Cancer Center, State Key Laboratory of Oncology in South China, Collaborative Innovation Center of Cancer Medicine, Guangzhou 510060, China

**Keywords:** EZH2, small molecule inhibitor, radiation therapy, chemotherapy, castration-resistant prostate cancer

## Abstract

Androgen deprivation therapy is the mainstay of treatment of advanced prostate cancer (PCa). However, a significant portion of patients experience disease relapse and tumors ultimately evolve into castration resistant prostate cancer (CRPC), for which there is no cure in the clinic. The Polycomb protein enhancer of zeste homolog 2 (EZH2) is frequently overexpressed in CRPC. It is unclear whether EZH2 can be a therapeutic target in CRPC. Here, we demonstrated that chemo- and radiotherapy agents such as camptothecin (CPT) and γ irradiation decrease EZH2 expression in various PCa cell lines. We provided evidence that functional p53 and RB proteins are required for CPT- and irradiation-induced downregulation of EZH2 in CRPC cells. We demonstrated that EZH2-specific small molecule inhibitors mitigate CRPC cell growth. We further showed that the EZH2 inhibitor GSK126 inhibits both Polycomb-dependent and -independent functions of EZH2 in PCa cells. Importantly, we found that inhibition of EZH2 by genetic and pharmacological means sensitizes CRPC cells to CPT-induced apoptotic death and growth inhibition in culture and in mice. Our data suggest that concomitant administration of small molecule inhibitors of EZH2 may significantly increase the anti-tumor efficacy of conventional chemo- and radiotherapies in CRPC.

## INTRODUCTION

Prostate cancer (PCa) is the most commonly diagnosed malignancy in men and the second leading cause of male cancer death in the United States. Androgen deprivation therapy (ADT) is the standard of care for patients with advanced/metastatic PCa. Unfortunately, this treatment is palliative, and 2–3 years after hormonal therapy most patients develop castration-resistant PCa (CRPC), from which patients eventually succumb.

The Polycomb group (PcG) protein EZH2 interacts with other PcG proteins such as SUZ12 and EED and forms a protein complex called Polycomb repressive complex-2 (PRC2). EZH2 functions as a methyltranstranferase in the PRC2 complex that promotes histone H3 lysine 27 trimethylation (H3K27me3) [1]. This histone modification promotes formation of repressive chromatin and causes epigenetic silencing of a large number of genes that drive cell differentiation and inhibit cell proliferation, migration and invasion [2–5].

Earlier studies show that the promoter activity of the *EZH2* gene is regulated by the transcription factor E2F1 and that EZH2 mRNA expression is regulated by the RB-E2F1 pathway [6]. Further studies demonstrate that expression of EZH2 is also regulated by sex hormones such as androgens and that this effect is mediated by p130, another pocket protein in the RB family and the transcription factor E2F4 [7]. In addition to regulation by transcription factors, EZH2 expression is also regulated by microRNAs such as miR101 [3].

Expression and function of EZH2 are often deregulated in PCa cells. The relevance of EZH2 in human prostate cancers is first evident by the finding that expression of EZH2 is highly upregulated in metastatic CRPC relative to the benign prostatic tissues and primary PCa [2]. Since this seminal discovery, interest in the crucial roles of EZH2 in PCa and other types of cancer is increasing exponentially [8–10]. EZH2 not only plays an essential role in anchorage-independent growth of PCa cells [9, 11], but is also required for PCa cell growth and invasion *in vitro* and metastasis in animals [3, 9, 11–14]. Moreover, it has been shown that AKT phosphorylates EZH2 at serine 21 and that this phosphorylation inhibits the Polycomb-dependent (PcD) function of EZH2 by blocking the assembling a functional PRC2 complex [15]. Importantly, it has been demonstrated that serine 21 on EZH2 becomes hyperphosphorylated in CPRC cells [16]. Hyperphosphorylation of EZH2 not only inhibits its H3K27me3-dependent gene repression function, but also renders EZH2 a Polycomb-independent (PcI) gene activation function in CRPC cells [16]. Notably, this function of EZH2 still depends on the methyltransferase activity [16]. Thus, EZH2 is not only overexpressed, but also gains new functions in CRPC cells, implying that it is a viable therapeutic target of CRPC.

Because of the deregulation of EZH2 in human PCa and many other cancer types, it becomes an ideal target for drug development. A number of EZH2 small molecule inhibitors have been developed and their antitumor efficacy has been tested in a number of tumor models such as lymphoma [17, 18]. However, their inhibitory effects on the PcI function of EZH2 and CRPC cell growth have not been tested. In the present study, we demonstrated that expression of EZH2 protein is downregulated by treatment of PCa cells with the chemotherapeutic agent camptothecin (CPT) and irradiation. This effect was primarily dependent on the activation of the p53 and RB pathways. We further showed that treatment of EZH2 inhibitors induces apoptosis of CRPC cells and this effect is largely enhanced by co-treatment of cells with CPT.

## RESULTS

### Inhibition of EZH2 expression by chemo- and radiotherapy agents in PCa cells

**Figure 1:**
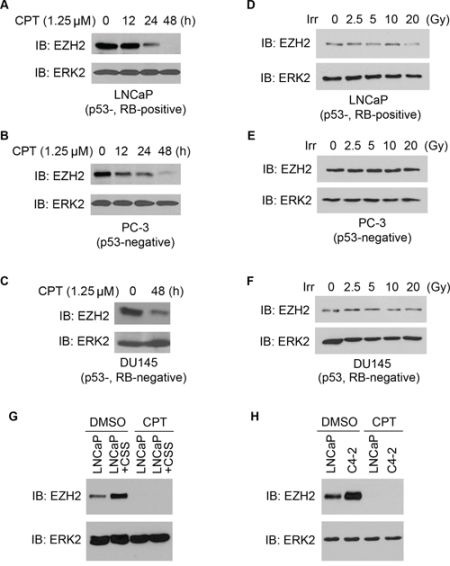
Treatment of PCa cells with chemo- and radiotherapy agents downregulates EZH2 expression **A–C.** effect of CPT on expression of EZH2 protein in PCa cells. LNCaP (p53-, RB-positive), PC-3 (p53-negative) and DU145 (p53-, RB-negative) PCa cell lines were treated with CPT (1.25 μM) and at different time points after treatment, cells were harvested for western blot analysis with indicated antibodies. ERK2 was used as a loading control. **D–F.** effect of γ irradiation on expression of EZH2 protein in PCa cells. LNCaP, PC-3 and DU145 cells were treated with different doses (Gray, Gy) of γ irradiation. At 24 h after treatment cells were harvested for western blot analysis using the indicated antibodies. Irr, irradiation. **G.** LNCaP cells were cultured in regular medium or medium containing charcoal-stripped-serum (CSS) for 48 h. Cells were then treated with CPT (1.25 μM) for 48 h and harvested for western blot analysis. **H.** LNCaP cells were cultured in regular medium and C4-2 cells were cultured in CSS medium for 48 h. Cells were then treated with CPT (1.25 μM) for 48 h and harvested for western blot analysis. ERK2 was used as a loading control.

Because expression of EZH2 is regulated by the RB/p130-E2F axis [6, 7] and this pathway is directly under the control of cyclin-dependent kinases (CDKs), we hypothesized that EZH2 expression can be inhibited due to the activation of the DNA damage-responsive pathways, which often leads to inhibition of CDKs [21]. To test this hypothesis, we treated three different PCa cell lines LNCaP, PC-3 and DU145 with camptothecin (CPT), a chemotherapeutic drug that inhibits the religation activity of topoisomerase-1 and therefore causes DNA double-strand breaks. We found that CPT treatment invariably decreased EZH2 protein expression, but to various extents in these cell lines (Figure 1A, 1B and 1C). By 48 h after CPT treatment, none, little and significant amount of EZH2 proteins were detected in LNCaP (p53- and RB-positive), PC-3 (p53-negative and RB-positive) and DU145 (p53- and RB-negative) cells, respectively (Figure 1A, 1B and 1C). These data suggest that the intactness of the p53 and RB pathways is important for CPT-induced downregulation of EZH2 proteins in PCa cells. This concept is further supported by the studies using γ irradiation. Although not robust as CPT treatment, γ irradiation also decreased EZH2 protein expression, at least at a very high dose, in LNCaP cells, which express a wild-type p53 (Figure 1D). In contrast, γ irradiation had little or no effect on EZH2 expression in PC-3 and DU145 cells, both of which are p53-deficient (Figure 1E and 1F).

It has been shown previously that castration increases EZH2 expression in LNCaP xenograft tumors in mice [7]. In agreement with this observation, EZH2 levels were substantially upregulated in LNCaP cells when cultured in androgen depleted medium (Figure 1G). Importantly, CPT treatment completely inhibited androgen deprivation-induced increase in EZH2 expression (Figure 1G). Also, consistent with the previous finding [7], EZH2 expression was much higher in castration-resistant C4-2 cells compared to androgen-sensitive LNCaP cells (Figure 1H). However, CPT treatment completely inhibited EZH2 expression in both cell lines (Figure 1H). Thus, our data show that expression of EZH2 is regulated by the chemotherapeutic agents such as CPT and irradiation and that the effects of these treatments are substantially affected by the intactness of p53 and/or RB pathways in PCa cells. These data also suggest that CPT treatment can overcome androgen deprivation-induced upregulation of EZH2 in CRPC cells.

### p53 is required for irradiation-induced downregulation of EZH2 in PCa cells

**Figure 2:**
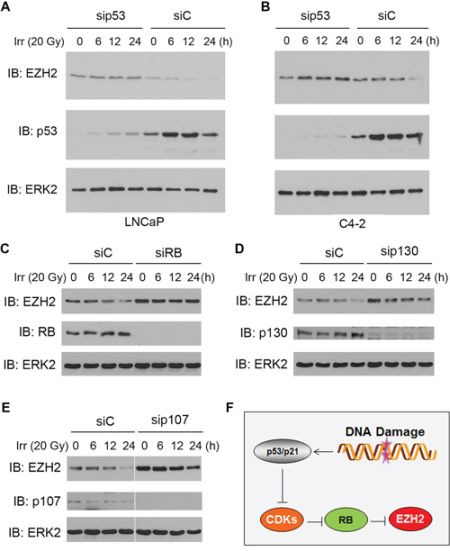
Role of p53 and the RB family proteins in irradiation-induced downregulation of EZH2 expression **A–B.** androgen-sensitive LNCaP (A) and castration-resistant C4-2 (B) cells were transfected with a pool of non-specific control siRNAs (siC) and p53-specific siRNAs. At 48 h after transfection cells were treated with γ irradiation, and cells were harvested at different time points for western blot analysis using the indicated antibodies. **C–E.** LNCaP cells were transfected with a pool of non-specific control siRNAs (siC) or RB-specific siRNAs (C), p130-specific siRNAs (D), or p107-specific siRNAs (E) At 48 h after transfection cells were treated with γ irradiation, and cells were harvested at different time points for western blot analysis using the indicated antibodies. ERK2 was used as a loading control. **F.** a hypothetical model depicting the causal role of p53 and RB in conventional chemotherapy (CPT) and radiotherapy (both are DNA damage-based therapeutics)-induced downregulation of EZH2 in PCa cells.

To determine the importance of a functional p53 in irradiation-mediated inhibition of EZH2 expression in PCa cells, endogenous p53 was knocked down by a pool of gene-specific siRNAs in androgen-sensitive LNCaP and castration-resistant C4-2 cells prior to irradiation treatment. As demonstrated in Figure 2A and 2B, endogenous p53 was effectively knocked down in both cell lines (at the 0-h time point of γ irradiation treatment). Importantly, depletion of p53 completely blocked irradiation-induced downregulation of EZH2 protein in both cell lines (Figure 2A and 2B). Thus, these results provide further support to the finding in p53-deficient PC-3 and DU145 cell lines (Figure 1E and 1F) that p53 is required for irradiation-induced downregulation of EZH2 protein in PCa cells.

### RB is important for irradiation-induced inhibition of EZH2 expression

Next, we examined the causal role of RB and other pocket proteins p107 and p130 in irradiation-induced suppression of EZH2 expression. To this end, endogenous RB, p107 and p130 was knocked down individually by a pool of gene-specific siRNAs in LNCaP cells, which express functional RB, p107 and p130 proteins [7]. Consistent with the previous reports that RB and p130 act as negative regulators of EZH2 [6, 7], knockdown of endogenous RB or p130 increased expression of EZH2 protein (Figure 2C and 2D). A similar result was obtained by knockdown of p107 (Figure 2E). As expected, treatment of LNCaP cells with γ irradiation decreased EZH2 protein levels, but this effect was completely abrogated by knockdown of endogenous RB (Figure 2C). In contrast, the effect of knockdown of p130 on irradiation-induced downregulation of EZH2 protein was very minimal compared to the effect of RB (Figure 2C and 2D). Similarly, knockdown of endogenous p107 failed to block irradiation-induced downregulation of EZH2 protein in these cells (Figure 2E). These data imply that the p53-CDK-RB signaling axis is important for irradiation-induced inhibition of EZH2 expression in PCa cells (Figure 2F).

### EZH2-specific small molecule inhibitors mitigate CRPC cell growth

**Figure 3:**
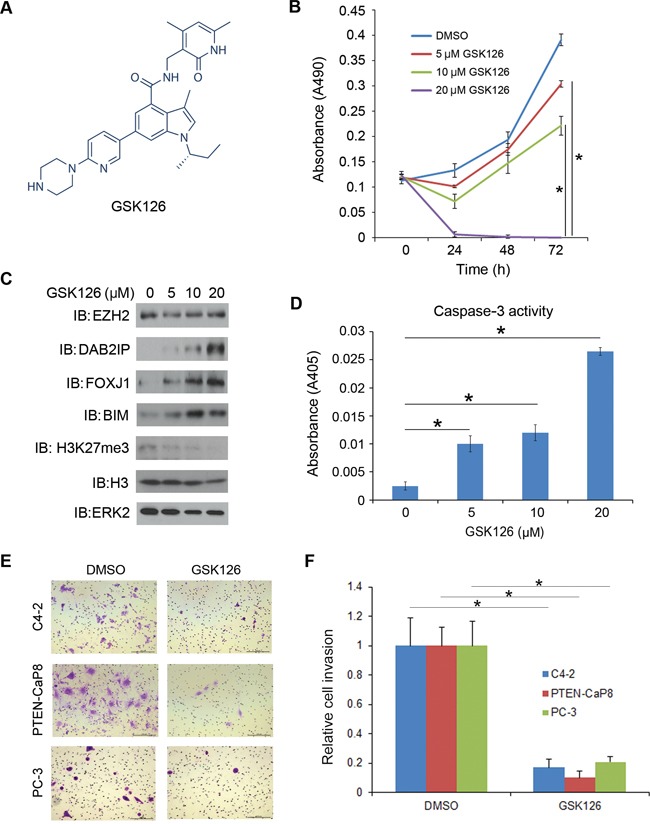
Inhibition of CRPC cell growth by the EZH2 small molecule inhibitor GSK126 **A.** The structure of GSK126 adapted from the website of Selleckchem. **B.** C4-2 CRPC cells were treated with the EZH2 specific inhibitor GSK126 with different doses (0, 5, 10, 20 μM). Cell viability was measured by MTS assays at different time points after GSK126 treatment. Data are means ± S.D. from six replicates. *P < 0.01. **C–D.** C4-2 CRPC cells were treated with the EZH2 specific inhibitor GSK126 with different doses (0, 5, 10, 20 μM). At 48 h after treatment cells were harvested for western blot analysis with indicated antibodies (C) and measurement of caspase-3 activity (D) ERK2 was used as a loading control. Data are means ± S.D. from 3 replicates. *P < 0.01. **E–F.** C4-2, PTEN-CaP8 and PC-3 cells were treated with vehicle (DMSO) or GSK126 (20 μM) for 48 h and then used for Matrigel invasion assays. Representative images of invasion assay are shown in (E) and the quantification results are shown in (F), respectively. Scale bar, 200 μm. Data are means ± S.D. from experiments with three replicates. *P < 0.01.

A series of EZH2-specific small molecule inhibitors including GSK126, GSK343 and GSK503 have been developed recently by GlaxoSmithKline and their effective anti-cancer activity has been demonstrated in various cancer models [17, 18, 22]. We have acquired these EZH2 inhibitors from GSK. Treatment of C4-2 CRPC cells with GSK126 (Figure 3A) decreased cell viability in a dose-dependent manner (Figure 3B). In agreement with this observation, treatment of GSK126 increased expression of BIM, a well-studied pro-apoptotic protein and caspase-3 activity (Figure 3C and 3D). The inhibition of the PcD function of EZH2 by GSK126 was evident by increased expression of known EZH2 targets DAB2IP and FOXJ1 [5, 23] and decreased levels of H3K27me3 (Figure 3C). Moreover, GSK126 treatment also decreased the number of C4-2, PTEN-CaP8 and PC-3 PCa cells invaded through the matrix (Figure 3E and 3F), although these data cannot rule out the possibility that the effect of GSK126 on cell invasion can be attributed, at least in part, to GSK126-induced cell death (Figure 3B and 3D).

**Figure 4:**
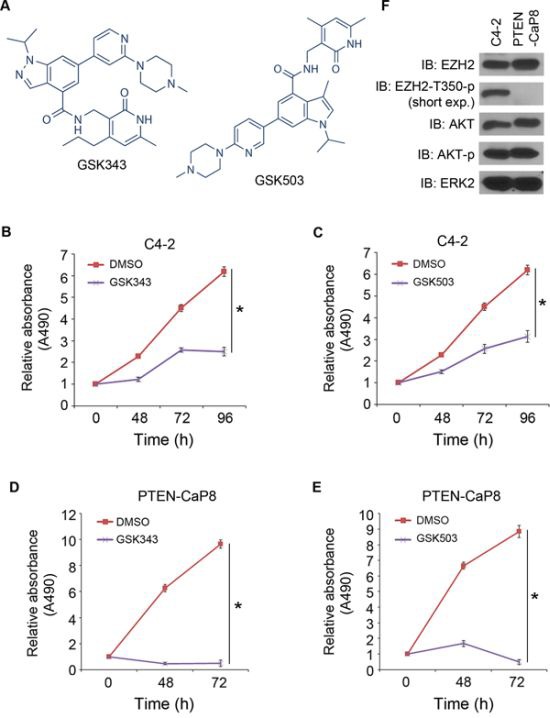
Inhibition of CRPC cell growth by the EZH2 small molecule inhibitors GSK343 and GSK503 **A.** The structures of GSK343 and GSK503 adapted from the website of Selleckchem. **B–E.** human C4-2 (B, C) and mouse PTEN-CaP8 (D, E) CRPC cells were treated with vehicle (DMSO), GSK343 or GSK503, two small molecule inhibitors of EZH2. At different time points, cell viability was measured by MTS assays. Data are means ± S.D. from six replicates. *P < 0.01. F, C4-2 and PTEN-CaP8 cells were harvested for western blot analysis of protein expression with indicated antibodies. ERK2 was used as a loading control. Short exp.: short exposure.

Treatment of C4-2 cells with other two EZH2 inhibitors GSK343 and GSK503 (Figure 4A) also decreased cell growth (Figure 4B and 4C). We also examined the anti-tumor effect of these EZH2 inhibitors on murine PTEN-null CRPC cell line PTEN-CaP8 [19]. Treatment with GSK343 and GSK503 also inhibited the growth of PTEN-CaP8 cells (Figure 4D and 4E). Intriguingly, the effect of these compounds was more pronounced in PTEN-CaP8 than in C4-2 cells (Figure 4B–4E). It has been showed previously that phosphorylation of threonine 350 on EZH2 is important for the functions of EZH2 in promoting H3K27me3 and gene silencing [5, 24] We demonstrated that EZH2 threonine 350 phosphorylation was substantially higher in C4-2 cells compared to PTEN-CaP8 cells (Figure 4F). We did not detect any overt difference in expression of total EZH2, total AKT and phosphorylated AKT (serine 473) between these two cell lines (Figure 4F). These data suggest that hypersensitivity of PTEN-CaP8 cells to the EZH2 inhibitors may be attributed to the lower activity of EZH2 in this cell line. Nevertheless, we demonstrated that different EZH2 inhibitors invariably inhibit the growth of both human and mouse CRPC cell lines, suggesting that EZH2 is a viable therapeutic target of CRPC.

### GSK126 inhibits Polycomb-dependent and -independent functions of EZH2 in PCa cells

**Figure 5:**
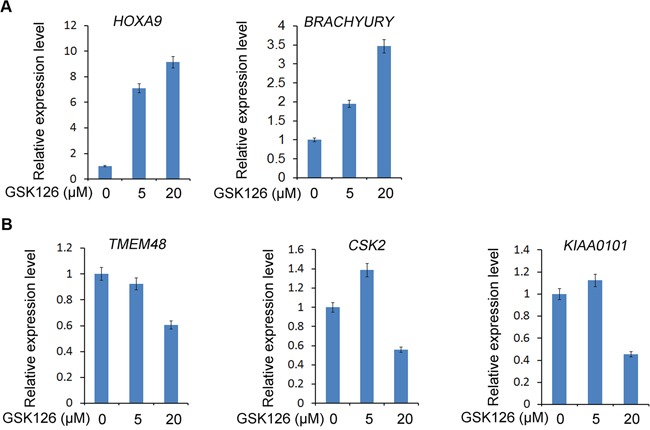
Effect of the EZH2 small molecule inhibitor GSK126 on expression of PcD and PcI genes in CRPC cells C4-2 CRPC cells were treated with different doses of GSK126. At 48 h after treatment, cells were harvested for RT-qPCR analysis of mRNA expression of EZH2-repressed target genes including *HOXA9* and *BRACHYURY* **A.** and EZH2-activated target genes including *TMEM48, CSK2* and *KIAA0101* **B.** *GAPDH* was used as an internal control.

In addition to canonical Polycomb-dependent (PcD) gene repression function, EZH2 also gains a Polycomb-independent (PcI) gene activation function in CRPC cells [16]. Given that both PcD and PcI functions of EZH2 are dependent on its methyltransferase activity [16], we sought to determine whether the EZH2 inhibitor can inhibit both PcD and PcI functions of EZH2 by affecting target gene expression. Consistent with the finding that treatment of C4-2 cells with GSK126 increased protein expression of the EZH2-repressed genes *DAB2IP* and *FOXJ1* (Figure 3C), GSK126 treatment also upregulated the expression of two other well-studied EZH2-repressed genes *HOXA9* and *BRACHYURY* (or called *T* gene) [5, 23] (Figure 5A). In agreement with the previous finding that EZH2 PcI target genes are well expressed in human CRPC cell lines C4-2B and CWR22Rv1 [16], we demonstrated that the PcI genes *TMEM48, CSK2* and *KIAA0101* were readily expressed in C4-2 CRPC cells (Figure 5B). Importantly, expression of these EZH2-activated genes was downregulated by GSK126, at least in a high dose (Figure 5B). Thus, both PcD and PcI functions of EZH2 can be inhibited by the EZH2 inhibitor GSK126 in human CRPC cells.

### The EZH2 inhibitor sensitizes CRPC cells to CPT-induced apoptotic death

**Figure 6:**
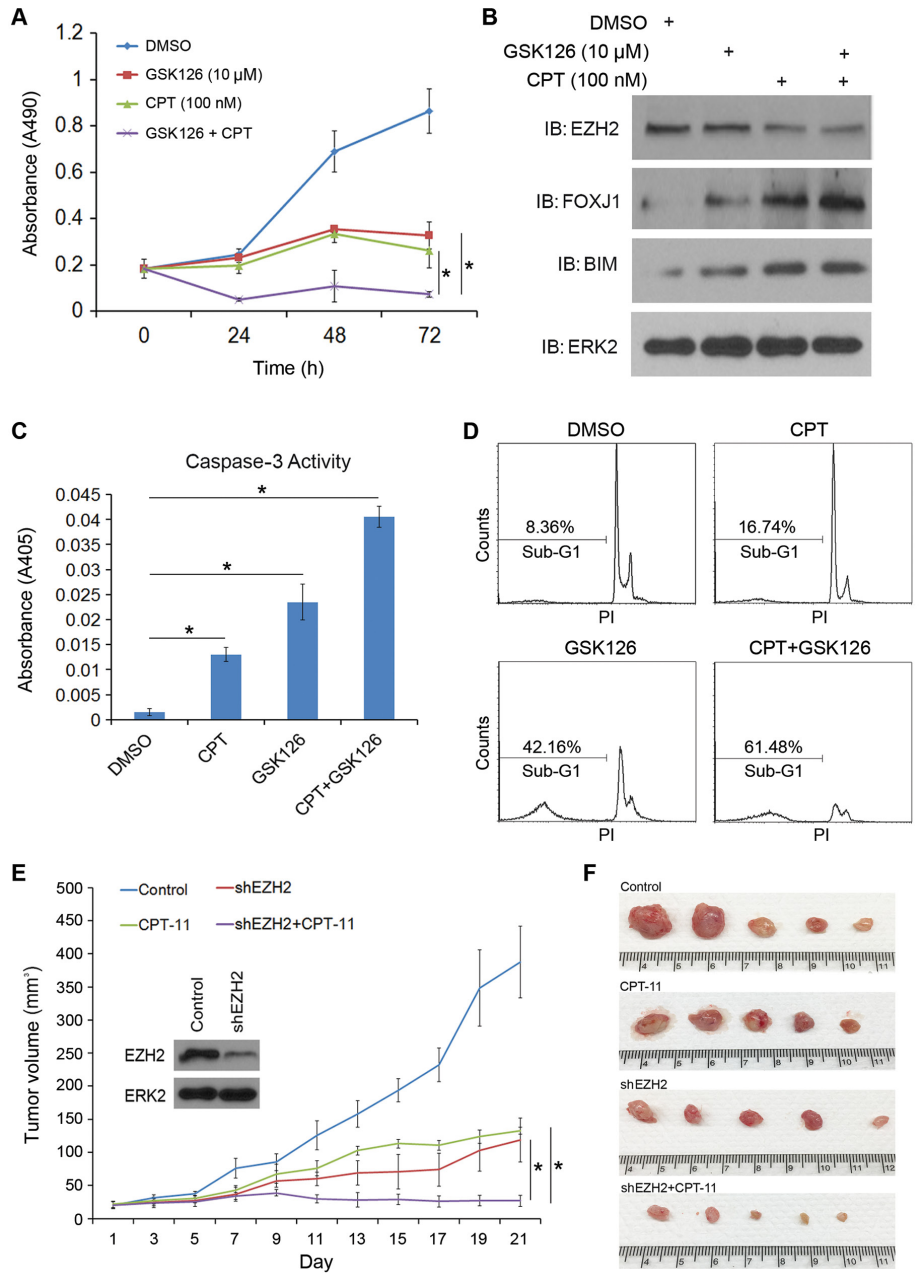
The EZH2 small molecule inhibitor GSK126 sensitizes CRPC cells to CPT-induced apoptotic death and enhances CPT-mediated inhibition of cell growth **A.** C4-2 CRPC cells were treated with vehicle (DMSO), CPT (100 nM), GSK126 (10 μM) or both drugs. Cell viability was measured at different time points after treatment using MTS assays. **B–C.** C4-2 CRPC cells were treated as in (A) At 48 h after treatment cells were harvested for western blot analysis with indicated antibodies (B) and measurement of caspase-3 activity (C) ERK2 was used as a loading control. Data are means ± S.D. from 3 replicates. *P < 0.01. **D.** C4-2 CRPC cells were treated with CPT (250 nM), GSK126 (20 μM) or both. At 48 h after treatment, cells were collected and fixed with 70% ethanol and subject to sub-G1 analysis flow cytometry. PI, propidium iodide. Experiments were repeated two more times and similar results were obtained. **E–F.** C4-2 cells infected with indicated shRNAs were injected s.c. into the right flank of NSG mice (*n* = 5). The tumor volume of each xenograft at each time point (E) and tumors at the end of treatment (F) are shown. Error bars, SD from five tumors. *P < 0.05.

Given that the chemotherapy agent CPT largely downregulates the level of EZH2 protein in p53-positive CRPC cells (Figure 1A), we sought to determine whether CPT treatment enables to sensitize CRPC cells to apoptotic death induced by the EZH2 inhibitor. To this end, we treated C4-2 CRPC cells with CPT alone or in combination with GSK126. As shown in Figure 6A, treatment with CPT or GSK126 alone decreased C4-2 cell viability. Intriguingly, an additive inhibitory effect was observed when cells were treated with both agents (Figure 6A). In agreement with these observations, co-treatment with CPT and GSK126 resulted in much greater induction of EZH2-repressed target FOXJ1 and the pro-apoptotic protein BIM in C4-2 cells (Figure 6B) as well as higher caspase-3 activity (Figure 6C). Moreover, FACS analysis indicated that the percentage of sub-G1 cells was much higher in CPT and GSK126 co-treated cells than in cells treated with each individual agent alone (Figure 6D). Furthermore, we demonstrated that combination of CPT treatment and shRNA-mediated knockdown of EZH2 resulted in much greater inhibition of C4-2 tumor xenograft growth in mice (Figure 6E and 6F). These data indicate that inhibition of EZH2 expression by chemotherapy agents and EZH2 activity by the small molecule inhibitors may have a greater therapeutic effect on CRPC cells.

## DISCUSSION

EZH2 is an oncogenic protein relevant to many, if not all, aspects of PCa, including cell proliferation, invasion and metastasis [2, 3]. Although significant progress has been made toward understanding the function and deregulation of EZH2 in PCa cells *in vitro* and prostate tumors *in vivo*, it remains largely unclear regarding how the novel regulatory mechanisms of EZH2 can be translated to effectively kill CRPC cells. In the present study we demonstrated for the first time that EZH2 expression is downregulated by the chemotherapeutic agent CPT and γ irradiation. Because expression of EZH2 is elevated following androgen deprivation in PCa cells in culture, in mice and in human patients [2, 7], our findings suggest that androgen deprivation-induced upregulation of EZH2 and associated oncogenic impacts on PCa cell function can be targeted by chemo- and radiotherapies. This notion is supported by our observation that CPT treatment abolishes androgen deprivation-induced increase in EZH2 protein levels in LNCaP and C4-2 cell models. Moreover, androgen deprivation therapy (ADT) combined with radiation therapy (RT) has become a mainstay of treatment for intermediate-to-high risk, locally advanced PCa [25–27]. The Radiation Therapy Oncology Group (RTOG) [28, 29], European Organization for Research and Treatment for Cancer (EORTC) [30] and others [31] have conducted randomized prospective trials and demonstrated an overall survival benefit in intermediate- and high-risk localized PCa when external beam radiation therapy (EBRT) is combined with ADT versus EBRT or ADT alone [32–34]. Thus, it is warranted to investigate whether irradiation-induced downregulation of EZH2 contributes to the therapeutic effect of ADT and RT in clinic. Furthermore, expression of EZH2 mRNA and protein are upregulated due to deletion of the PTEN tumor suppressor in PCa cells in culture and in mice [35–37]. Given that loss of PTEN occurs in approximately 20% of localized and over 60% of advanced/metastatic PCa in patients, it is also of paramount importance to explore in the future whether PTEN loss could serve as a molecular determinant for irradiation-induced downregulation of EZH2 protein and the associated therapeutic benefits in PCa patients.

An important aspect of our findings is that irradiation-induced downregulation of EZH2 is abolished by depletion of p53. The same is true in RB-deficient PCa cells. It is well documented that the *TP53* gene is frequently mutated in metastatic or CRPC samples [38, 39]. Also, a significant portion of human prostate cancers have loss of RB [38]. Therefore, it can be postulated that frequent loss of p53 and RB in PCa may cause the resistance to chemo- or radiotherapy-induced downregulation of EZH2 and thereby compromise the therapeutic effect of these regimens in a large segment of patients.

EZH2-specific inhibitors have been developed recently and their anti-cancer activity has been manifested in various cancer models including lymphomas [17, 40]. Because EZH2 is frequently overexpressed in human metastatic CRPC in patients [2], it is both relevant and significant to determine the therapeutic effect of EZH2 inhibitors on CRPC cells. Using both human and murine CRPC cell lines as working models, we demonstrated that treatment with different EZH2 inhibitors invariably induce a decrease in cell growth and viability. We further showed that this effect is attributed to apoptotic cell death. At present, it is unclear which downstream pathways mediate EZH2 inhibitor-induced apoptotic death of CRPC cells and further investigation is warranted. It is worth noting that in addition to the well-studied PcD gene repression function of EZH2, a PcI gene activation function of EZH2 has been uncovered recently [16]. In agreement with the findings that both PcD and PcI functions of EZH2 require the methyltransferase activity [16], we demonstrated that treatment of the EZH2 inhibitor GSK126 not only causes de-repression of EZH2-repressed genes such as *BRACHYURY, HOXA9, DAB2IP* and *FOXJ1*, but also downregulates the expression of EZH2-activated genes such as *TMEM48, CSK2* and *KIAA0101*. Thus, the anti-tumor effect of EZH2 inhibitors is likely attributed to their inhibition of both PcD and PcI functions of EZH2.

The anti-tumor effect of the EZH2 inhibitor GSK126 was detected in the lymphoma model at the concentrations of nanomoles. However, the effective concentrations of GSK126 in CPRC cells were much higher than those used in lymphoma models. Our finding that the level of EZH2 can be downregulated by the chemotherapeutic agent CPT suggests that co-treatment of PCa with CPT may increase the sensitivity of CRPC cells to the EZH2 inhibitor. Indeed, we provide evidence that CPT largely enhances cell death induced by GSK126 and co-administration of both drugs results in much greater inhibitory effect on CRPC cell growth. Together, our findings suggest that combined administration of small molecule inhibitors of EZH2 with the conventional chemotherapy and radiotherapy represents a new strategy for effective treatment of CRPC in patients.

## MATERIALS AND METHODS

### Cell lines, cell culture and reagents

LNCaP, PC-3 and DU145 PCa cell lines were purchased from American Type Culture Collection (Manassas, VA) in 2010. C4-2 CRPC cell line was purchased from Uro Corporation (Oklahoma City, OK) in 2006. PTEN-CaP8 murine PTEN-deficient CRPC cell line was kindly provided by Dr. Hong Wu at UCLA [19]. Cells were cultured in RPMI 1640 containing 10% fetal bovine serum (Life Technologies) and 100 units/ml penicillin and 100 μg/ml streptomycin (Life Technologies). All cell lines were cultured at 5% CO_2_, 37°C and 95% humidity. Authentication of these cell lines was performed in our laboratory within 6 months of this submission by examining the expression of epithelial cell markers, androgen receptor (AR), prostate-specific antigen (PSA), PTEN, total AKT and AKT phosphorylation at serine residues 473 and 308 using western blots. Camptothecin (CPT) was purchased from Sigma-Aldrich. The EZH2 small molecule inhibitors GSK126, GSK343 and GSK503 [17, 18] were kindly provided by GlaxoSmithKline (GSK). Non-specific control and EZH2-specific small hairpin RNAs (shRNAs) were purchased from Sigma-Aldrich.

### Western blot analysis

Cells were lysed in modified RIPA buffer [1 × PBS, 1% Nonidet P-40, 0.1% sodium dodecyl sulfate and protease inhibitor cocktail (Sigma-Aldrich)]. The concentration of samples was measured by BCA assay (Thermo Fisher Scientific). Equal amounts of samples were separated by 6–10% SDS-polyacrylamide gels and transferred to nitrocellulose membranes. Subsequently, membranes were incubated with primary antibody overnight at 4°C. Next day, the membranes were washed with 1 × TBST and incubated in room temperature with HRP-conjugated secondary antibody for 1 h. Proteins were detected by chemiluminescence. HRP-conjugated secondary antibodies were purchased from GE Healthcare. The following antibodies were used: EZH2, RB, histone H3, AKT and phosphor-AKT (serine 473) (Cell Signaling Technology); p53, p130 and p107 (Santa Cruz Biotechnology); FOXJ1 and BIM (Millipore); DAB2IP and H3K27me3 (Abcam). EZH2 threonine 350 phosphorylation-specific antibodies were generated as we reported previously [5].

### RNA extraction and real-time PCR

Total RNA was extracted from cultured cells using Trizol reagent (Life Technologies). cDNA was synthesized using Superscript II reverse transcriptase (Life Technologies). Real-time PCR was performed using IQ SYRB Green Supermix and an iCycler iQTX detection system (Bio-Rad). All the signals were normalized by *GAPDH* and the 2-ΔΔCt method was used to determine the fold changes. Primers used were as follows: *BRACHYURY*, forward 5′-AGGTGGGGAAGTTTCCTTCT-3′ and reverse 5′-GCAAATGAGGTCCTTTTGGT-3′; *HOXA9*, forward 5′-TTGGAGGAAATGAATGCTGA-3′ and reverse 5′-TGGTCAGTAGGCCTTGAGGT-3′; *TMEM48*, forward 5′-AGGTCGCGGGACATACTGT-3′ and reverse 5′-TGCAGATGGGTAGAAATAGCACT-3′; *CSK2*, forward 5′-TTCGACGAACACTACGAGTACC-3′ and reverse 5′-GGACACCAAGTCTCCTCCAC-3′; *KIAA0101*, forward 5′-ATGGTGCGGACTAAAGCAGAC-3′ and reverse 5′-CCTCGATGAAACTGATGTCGAAT-3′; *GAPDH*, forward 5′-ACCCACTCCTCCACCTTTGAC-3′ and reverse 5′-TGTTGCTGTAGCCAAATTCGTT-3′.

### Small interference RNA (siRNA)

Pools of non-specific control siRNAs (siC) or gene-specific siRNAs for p53, RB, p107 and p130 were purchased from GE Health Dharmacon. Transfections were performed by electroporation using an Electro Square Porator ECM 830 (BTX) or by using Lipofectamine 2000 (Life Technologies). Approximately 75–90% transfection efficiencies were routinely achieved.

### Cell invasion assay

*In vitro* cell invasion assay was performed using BioCoat Matrigel invasion chamber (BD Biosciences) according to the manufacturer's protocol. C4-2, PTEN-CaP8 and PC-3 cells were cultured in the insert for 24 h. Cells were fixed in methanol for 15 min and then stained with 1 mg/ml crystal violet for 20 min. At least 5 fields for each group were photographed after staining and invaded cells were counted.

### MTS assay

C4-2 and PTEN-CaP8 cells (5 × 10^3^) were plated into 96-well plates and cultured in medium containing 10% FBS. At 24 h after plating, cells were treated with EZH2 inhibitors and MTS solution (Promega) was added into each well. At different time points (from 0 to 96 h after drug treatment), plates were incubated for 2 h at 37°C. Absorbance at a wavelength of 490 nm (A490) was measured with a Versamax Microplate Reader. Growth curve was calculated according to the values of A490 of six replicates at each time point.

### Analysis of apoptotic cells using fluorescence-activated cell sorting (FACS)

Apoptotic cell death (sub-G1) was analyzed using FACS as we described previously [20]. C4-2 cells were treated with vehicle (DMSO), CPT, GSK126 or both. At 48 h after treatment, cells were collected and washed with 1 × phosphate-buffered saline (PBS). After fixation with 70% ethanol, cells were washed twice with 1 × PBS and stained with a solution containing 20 μg/ml propidium iodide and 50 μg/ml RNase A. Cells were incubated for 30 min at the room temperature and cell cycle profiles were determined by flow cytometry using a FACScan (Becton-Dickinson).

### Caspase-3 activity measurement

The activity of caspase-3 was measured by Caspase-3 Colorimetric Protease Assay (Life Technologies). Briefly, cells were resuspended in 50 μl lysis buffer and incubated on ice for 10 min. Cellular proteins (100 μg) were diluted in 50 μl lysis buffer and 50 μl reaction buffer (containing 10 mmol/L DTT). Five microliters of the 4 mmol/l DEVD-*p*NA substrate (200 μmol/l final concentration) were added, and the reaction was carried out at 37°C for 2 h in the dark. Reactions were measured in a microplate reader at 405 nm (A405).

### Generation and treatment of PCa xenografts in mice

Six-week-old NOD-SCID IL-2-receptor gamma null (NSG) mice were generated in house and used for animal experiments. The animal study was approved by the Mayo Clinic IACUC. All mice were housed in standard conditions with a 12 h light/dark cycle and access to food and water ad libitum. C4-2 cells (3 × 10^6^) infected with lentivirus expressing control shRNA (control) or EZH2 shRNA were injected s.c. into the right flank of mice. When xenografts were well established (7 days after injection), mice were treated i.p. with CPT-11 (10 mg/kg/day). The tumor size was measured every other day for 21 days and calculated using the formula LxW^2^x0.5.

### Statistical analysis

Experiments were carried out with three or more replicates unless indicated specifically. Statistical analyses were performed by two-tailed Student's t test. *P* < 0.05 is considered statistically significant.

## Abbreviations

PCa: prostate cancer
CRPC: castration-resistant prostate cancer
EZH2: zeste homolog 2
CPT: camptothecin
PRC2: Polycomb repressive complex 2
H3K27me3: histone H3 lysine 27 trimethylation
NSG: NOD-SCID IL-2-receptor gamma null
CDK: cyclin-dependent kinases
PcD: Polycomb-dependent
PcI: Polycomb-independent
